# The association of nuclear abnormalities in exfoliated buccal epithelial cells with the health status of different agricultural activities farmers in Peninsular Malaysia

**DOI:** 10.1186/s41021-016-0032-1

**Published:** 2016-03-01

**Authors:** Zariyantey Abdul Hamid, Mohd Faizal Mohd Zulkifly, Asmah Hamid, Syarif Husin Lubis, Nihayah Mohammad, Ismarulyusda Ishak, Nur Zakiah Mohd Saat, Hidayatul Fathi Othman, Ahmad Rohi Ghazali, Mohd Jamil Mohd Rafaai, Mohamad Roff Mohd Noor, Nor Fadilah Rajab

**Affiliations:** Biomedical Science Program, School of Diagnostic and Applied Health Sciences, Faculty of Health Sciences, Universiti Kebangsaan Malaysia (UKM,), Jalan Raja Muda Abdul Aziz, Kuala Lumpur, 50300 Malaysia; Horticulture Research Centre, MARDI, Persiaran MARDI-UPM, Serdang, 43400 Selangor Malaysia; Center for Neuroscience Services and Research (P3Neuro), Universiti Sains Malaysia, 16150 Kubang Kerian, Kota Baru, Kelantan Malaysia

**Keywords:** Pesticides, Farmers, Micronucleus, Binucleus, Buccal cells

## Abstract

**Background:**

Pesticide exposure possesses risk of genotoxicity to humans, particularly farmers. Despite accumulating evidences linking genotoxicity to pesticide exposure, epidemiological studies to address pesticide toxicity in occupationally exposed farmers in Malaysia remain underreported. Thus, this study was aimed to determine the presence of nuclear abnormalities through the assessment of micronucleus (MN) and binucleus (BNu) frequencies in exfoliated buccal epithelial cells from farmers who were exposed to pesticides. A cross-sectional study of farmers among different agricultural activities farmers in Bachok and Pasir Puteh, Kelantan, North East of Peninsular Malaysia was done to evaluate the presence of nuclear abnormalities and its correlation with their health status and farming activities.

**Results:**

Analysis of buccal cells revealed that the frequency of MN was significantly higher (*p* < 0.05) in farmers as compared to controls. In contrast, no significant difference (*p* > 0.05) was observed for BNu frequency in between groups. Correlation analysis showed that apart from a significant (*p* < 0.05) and positive correlation between the duration of fertilizers exposure and frequencies of MN (*r* = 0.42, *P* = 0.001) and BNu (*r* = 0.37, *P* = 0.02), no other correlation of various confounding factors on the formation of MN and BNu were observed.

**Conclusion:**

In conclusion, pesticide and fertilizers exposure may contribute to the promotion of nuclear anomalies among Malaysian farmers who are engaged in mixed plantation activities. Further assessment of larger populations is important to address and overcome the potential risk of pesticide-induced genotoxicity.

## Background

Worldwide, extensive use of pesticides in the agriculture sector can produce substantial threats to the environment and human health. Pesticides contain a great variety of components that differ in their composition and properties, with many that were classified as carcinogenic by the International Agency for Research on Cancer [1]. In the context of occupational exposure, farmers are the most at risk to health related-pesticide toxicity [2]. Long-term exposure to pesticides has been associated with a number of human health effects such as hormonal disruption, reproductive abnormalities, cancer, neurological disorders and cardiorespiratory symptoms [3–6].

The ability of pesticides to induce DNA damage has been reported in a number of studies [7–10] and a positive correlation between pesticides exposure and the genotoxicity risk has been previously documented [8, 11]. The findings suggested that exposure to pesticide can be genotoxic and carcinogenic to humans [12] which impose a need for genotoxicological biomonitoring in human populations who are at greater risk of pesticide-mediated toxicity.

Human biological monitoring offers a useful applied approach to evaluate potential genotoxic risk associated with exposure to various environmental genotoxicants. Cytogenetic damage such as formation of micronucleus (MN), chromosomal aberration (CA) and sister chromatid exchange (SCE) has been widely used as biomarkers to indicate possible genotoxic risk of a defined exposure [13, 14]. Among these, micronucleus testing is the most preferred approach due to it being less laborious, cheap and easy to apply in epidemiological studies [15, 16].

Evidences for increase in MN frequency in human population exposed to pesticide have been reported in several studies [17–19]. Cytogenetic monitoring is a widely accepted tool to estimate the genetic damage associated with pesticide exposure as is evident from its application in numerous epidemiological studies conducted to date [20–23].

MN is known as a product of acentric chromosomal fragments or fragments of the whole chromosomes left during mitotic cellular division which represent structural or numerical chromosomal aberrations (or both) during mitosis [24, 25]. In comparison, binucleated cells (BNu) is defined as the presence of two nuclei that are adherent to each other and can indicate failure in cytokinesis [26]. Together, these biomarkers can allow detection of nuclear anomalies which indicate potential risk of pesticide-mediated genotoxicity. Sampling of exfoliated buccal epithelial cells were preferred due to its less laborious sample collection technique, a non- invasive approach and a sensitive method to monitor genetic damage among human populations [27, 28].

While pesticide exposure is known for its potential toxicity effects, epidemiological studies to address pesticide toxicity in occupationally exposed farmers in Malaysia remain underreported. Thus, this study is aimed to determine the presence of nuclear abnormalities through the assessment of MN and BNu frequencies in exfoliated buccal epithelial cells from farmers who were exposed to pesticides in Bachok and Pasir Puteh, Kelantan, North East of Peninsular Malaysia. Influence of various confounding factors which include the use of personal protective equipment (PPE), type of agriculture activities and demographic status on the frequencies of MN and BNu were also considered. Together, this study can provide information on the safety aspect of farming activities in Malaysia in order to promote appropriate pesticide handling practices in agriculture sectors.

## Methods

### Study design

A cross-sectional study was conducted in the East Coast of Peninsular Malaysia. The sample frame was a list of farmers as registered by the District Farmers Association of Bachok and Pasir Puteh. Systematic random sampling method was employed to recruit farmers and sample size estimation was determined using the method described in Cochran [29] based on the standard deviation from a study conducted by Pastor et al. [30]. The inclusion criteria for subjects’ selection were farmers aged between 19 to 60 years old who have been working as a farmer for a minimum of 1 year and have never stopped working for more than 3 months in a year. The exclusion criteria were farmers who have a family history of cancer or have been diagnosed for any cancer. A structured questionnaire was used to gather information on socio-demographics, knowledge on safe pesticides handling practices, pesticides application and the use of personal protective equipment (PPE). The study was explained to the potential subjects and informed consent was obtained prior to buccal cell sampling. The questionnaire was administered face-to-face. The protocol of this study was approved by the Universiti Kebangsaan Malaysia Ethics Committee (UKMEC) (UKM1.5.3.5/244/NN-201-2010). This study was conducted as part of a collaborative research with the Malaysian Agriculture and Research Development Institute (MARDI) on the health impact associated with pesticides exposure among farmers in Malaysia.

### Subjects

The study was carried out on 39 farmers in Bachok (*n* = 11) and Pasir Puteh (*n* = 28), Kelantan. The areas were selected for its extensive use of pesticides and fertilizers with mixed plantation activities. The main crops grown were rice, tobacco and varieties of vegetables such as chillies, cucumbers and tapiocas. The control subjects have no record of pesticide exposure and were composed of 30 office staff from UKM Jalan Raja Muda Abdul Aziz campus, Federal Territory of Kuala Lumpur, Malaysia.

### Buccal cells collection

Collection of buccal cells was performed according to the method as described in [30] with minor modifications. Briefly, prior to sample collection, subjects rinsed their mouth with water for removal of unwanted contaminants. Buccal cells were obtained by rubbing the buccal mucosa layer using a sterile wooden tongue depressor. The buccal cells were washed three times in 5 ml buffered solution (0.003 M EDTA, 0.01 M Tris–HCl, 0.64 M NaCl) at pH 7.0 by centrifugation at 2000 rpm for 10 min each, with a brief vortexing at every interval for dissociation of the cell pellets. After the third wash, cell pellets were added with 5 ml of 0.075 M KCL and 50 μl of 1 % DMSO. The mixtures were incubated for 30 min at room temperature followed by the addition of 1 ml of Carnoy fixative solution (methanol and acetic acid at a ratio of 3:1). The mixtures were then centrifuged at 2000 rpm for 10 min. Following removal of the supernatant, the cell pellets were fixed with 10 ml Carnoy fixative solution and stored at -20 °C until further analysis.

### Evaluation of micronuclei and binucleated buccal cells

Fixed cell pellets were washed twice in 10 ml Carnoy fixative solution at 2000 rpm for 10 min. At the second wash, most of the supernatants were discarded except a leftover solution of approximately 1 ml. 200 μl of cell suspensions was dropped onto preheated (37 °C) and pre-cleaned frosted slides. The slides were allowed to air-dry for 5–10 min and stained with 0.0025 % acridine orange (AO) for microscopic analysis. Cytological slides were prepared in triplicates for each subject. A total of 1000 cells were counted for each subject and the frequency of MN and BNu were determined using a fluorescence microscope (OLYMPUS XC50) at x200 and x400 magnifications.

### Stastical analysis

Data were analysed using the SPSS statistical software package version 18.0. The level of significance was set as *p* ≤0.05. Data were tested for normality using the Shapiro-Wilk test. Student’s *t*-test was used for the analysis of differences between groups. The correlation between the two variables was analyzed using Pearson correlation.

## Results

The demographic data of the studied population (Table 1) indicate that the majority of the farmers in Bachok and Pasir Puteh (56.4 %) were aged between 56 to 65 years old while in the control group, the majority of subjects (40.0 %) were aged between 36 to 45 years old. Meanwhile, no remarkable differences were observed for both groups with regards to other general characteristics, which include educational background, smoking status and body mass index (BMI). The majority of farmers (F) and control (C) were pre-obese (*F* = 51.3 %; *C* = 50.0 %), completed secondary school (*F* = 51.3 %; *C* = 63.3 %) and non-smokers (*F* = 56.4 %; *C* = 70.0 %).

**Table 1:** General characteristics of studied population

Characteristics	Farmers	Controls
	*n* = 39(%)	*n* = 30(%)
*Age groups*		
26–35	2(5.1)	6(20.0)
36–45	7(17.9)	12(40.0)
46–55	8(20.5)	10(33.3)
56–65	22(56.4)	2(6.7)
*Gender*		
Male	33(84.6)	21(70.0)
Female	6(15.4)	9(30.0)
*Education*		
None	2(5.1)	0(0)
Primary school	17(43.6)	2(6.7)
Secondary school	20(51.3)	19(63.3)
University	0(0)	9(30.0)
*Smoking*		
Smokers	17(43.6)	9(30.0)
Non-smokers	22(56.4)	21(70.0)
*BMI (kg/m* ^*2*^ *)*		
Underweight *(<18.5)*	4(10.3)	0(0)
Normal *(18.5–24.9)*	13(33.3)	9(30.0)
Pre-obese *(25.0–29.9)*	20(51.3)	15(50.0)
Obese grade 1 *(30.0–34.9)*	2(5.1)	3(10.0)
Obese grade 2 *(35.0–39.9)*	0(0)	3(10.0)

With respect to chronic diseases and health-associated symptoms (Table 2), farmers had higher percentages of hypertension (30.8 %), asthma (20.5 %) and gastric (28.2 %) as compared to that observed in the control group. Additionally, more than 50 % of farmers claimed to frequently experience blurred vision and numbness. Other symptoms such as eye irritation, coughing, insomnia and runny nose were also found to be higher in farmers as compared to control.

**Table 2:** Commonly reported chronic diseases and health symptoms

	Farmers	Controls
	*n* = 39(%)	*n* = 30(%)
*Chronic Diseases*		
Hypertension	12(30.8)	5(16.7)
Diabetes Mellitus	2(5.1)	3(10.0)
Asthma	8(20.5)	2(6.7)
Gastric	11(28.2)	3(10.0)
*Health-associated symptoms*		
Sore throat	7(17.9)	3(10.0)
Headache	14(35.9)	10(33.3)
Blurred vision	22(56.4)	10(33.3)
Insomnia	6(15.4)	1(3.3)
Eyes irritation	17(43.6)	7(23.3)
Runny nose	6(15.4)	1(3.3)
Numbness	23(59.0)	2(6.7)
Cough	17(43.6)	4(13.3)

With regards to the application of personal protective equipments (PPE), a majority of farmers (Table 3) claimed the use of safety helmets (89.7 %), long-sleeved shirt (89.7 %), long pants (97.4 %), nose mask (84.6 %) and boots (71.8 %) during pesticide handling. Meanwhile, usage of gloves was at a moderate level with only 53.8 % who claimed to use it. Eye goggles were found to be the least used PPE with 69.2 % of farmers who claimed that they never used eye goggles while handling pesticides.

**Table 3:** Application of personal protective equipment (PPE) among farmers

PPE	*n* = 39(%)
*Safety helmets*	
Yes	35(89.7)
No	4(10.3)
*Long-sleeve shirts*	
Yes	35(89.7)
No	4(10.3)
*Long Pants*	
Yes	38(97.4)
No	1(2.6)
*Eye goggles*	
Yes	12(30.8)
No	27(69.2)
*Nose mask*	
Yes	33(84.6)
No	6(15.4)
*Gloves*	
Yes	21(53.8)
No	18(46.2)
*Boots*	
Yes	28(71.8)
No	11(28.2)

Additional information with regards to farming activities were also evaluated. The information includes type of crops, farming experiences, duration of pestiside and fertilizers exposure and types of pesticide applicators being utilized. As shown in Table 4, the main crops grown were paddy, vegetables and tobacco. With respect to plantation activities, 36 % of farmers involved in vegetable farming while about 25.6 % were paddy and tobacco farmers. In addition, 12.8 % of farmers involved with mixed farming consisted of paddy, vegetables and tobacco. The majority of farmers (28.2 %) have been farming in between 1 and 10 and 11–20 years. Moreover, majority of farmers (64.1 %) had pesticide spraying frequencies of more than 3 times in a month with most of them (53.8 %) spent between 1 to 12 h per month for such activities. As for fertilizers exposure, most of farmers (61.5 %) spent about 1 to 4 h on fertilizers usage per month with majority (76.9 %) exposed to fertilizers between 1 to 3 times per month. With regards to the type of pestiside applicators, farmers mostly used motorized knapsack sprayer (74.4 %) as compared to other methods. Meanwhile, with regards to common personal habits during and / or after pestiside exposure, majority of farmers (>80 %) showed a satisfactory practice on this aspect (Table 5).

**Table 4:** Agriculture activities among farmers

Activities	Farmers
	*n* = 39(%)
*Crops*	
Vegetables	14(36.0)
Paddy	10(25.6)
Tobacco	10(25.6)
Combination of vegetables, paddy and tobacco	5(12.8)
*Farming experiences*	
Number of years	
1–10	11(28.2)
11–20	11(28.2)
21–30	8(20.5)
31–40	3(7.7)
41–50	6(15.4)
*Frequencies of pesticides spraying and application of fertilizers*	
Frequency per month	
Pesticides	
1–3 times	14(35.9)
>3 times	25(64.1)
Fertilizers	
1–3 times	30(76.9)
>3 times	9(23.1)
*Duration of pesticides spraying and application of fertilizers*	
Spent hours per month	
Pesticides	
1–12 h	21(53.8)
>12 h	18(46.2)
Fertilizers	
1–4 h	24(61.5)
>4 h	15(38.5)
*Pesticides application equipments*	
Motorized knapsack sprayer	29(74.4)
Mechanical sprayer	5(12.8)
Other sprayer	5(12.8)

**Table 5:** Personal habits during and/or after pesticides exposure

Personal habits	Farmers
	*n* = 39(%)
*Personal habits during pesticide applications*	
Drinking	
Yes	6(15.4)
No	33(84.6)
Eating	
Yes	5(12.8)
No	34(87.2)
Smoking	
Yes	3(7.7)
No	36(92.3)
*Post-pesticides application practices*	
Change clothes	
Yes	34(87.2)
No	5(12.8)
Wash hand	
Yes	37(94.9)
No	2(5.1)

The mean ± S.D of micronucleus (MN) and binucleus (BNu) per 1000 cells of farmers and controls are as presented in Table 6. The frequency of MN was found to be significantly higher (*p* < 0.05) among farmers (6.83 ± 6.25) as compared to controls (1.39 ± 0.60). However, no significant difference was observed for the frequency of BNu between both groups.

**Table 6:** Frequencies of MN and BNu in farmers and controls according to age, gender, smoking status, BMI and duration of employment. Data are presented as mean ± standard deviation (s.d)

	Controls (*n* = 30)		Farmers (*n* = 39)	
	Mean ± S.D		Mean ± S.D	
Groups	Frequency of MN (%)	Frequency of BNu (%)	Frequency of MN (%)	Frequency of BNu (%)
*Age (years)*				
26–35	1.01 ± 0.32	2.01 ± 0.73	6.65 ± 0.92*	1.50 ± 1.41
36–45	1.47 ± 0.58	1.67 ± 0.94	6.22 ± 1.90*	1.51 ± 0.19
46–55	1.57 ± 0.72	1.49 ± 0.86	8.01 ± 3.09*	1.33 ± 0.45
56–65	1.10 ± 0.14	1.85 ± 0.64	6.61 ± 3.21*	1.63 ± 0.74
*Gender*				
Male	1.32 ± 0.55	1.85 ± 0.86	6.75 ± 3.01*	1.49 ± 0.61
Female	1.56 ± 0.70	1.32 ± 0.70	7.23 ± 2.43*	1.80 ± 0.83
*Smoking status*				
Smokers	1.25 ± 0.41	1.99 ± 1.08	6.82 ± 2.65*	1.39 ± 0.62
Non-smokers	1.45 ± 0.66	1.56 ± 0.71	6.83 ± 3.15*	1.65 ± 0.65
*BMI*				
Underweight	-	-	6.89 ± 3.04	1.49 ± 0.80
Normal	1.54 ± 0.61	1.59 ± 1.19	7.83 ± 3.19*	1.43 ± 0.77
Pre-obese	1.22 ± 0.47	1.72 ± 0.62	6.22 ± 2.58*	1.58 ± 0.57
Obese grade 1	1.66 ± 0.31	1.30 ± 0.61	6.25 ± 4.74	1.85 ± 0.35
Obese grade 2	1.57 ± 1.25	2.23 ± 0.91	-	-
*Duration of employment (years)*				
1–10	1.39 ± 0.50	1.74 ± 0.99	7.04 ± 2.39*	1.34 ± 0.61
11–20	1.21 ± 0.64	1.74 ± 0.72	6.14 ± 3.02*	1.54 ± 0.42
21–30	1.67 ± 0.83	1.56 ± 0.93	5.80 ± 2.06*	1.76 ± 0.48
31–40	1.25 ± 0.53	1.63 ± 0.52	7.32 ± 4.20*	1.29 ± 0.88
41–50	-	-	8.82 ± 3.71	1.71 ± 1.08
*Total*	1.39 ± 0.60	1.69 ± 0.84	6.83 ± 2.91*	1.54 ± 0.64

*Farmers versus controls (*p* < 0.05)

The intra- (within) groups comparison of MN and BNu frequencies for age, gender, smoking status, BMI and duration of employment showed no significant difference. In contrast, inter—(between) groups comparison revealed a significantly higher (*p* < 0.05) frequency of MN among farmers as compared to control at every age group. Similarly, a significantly higher (*p* < 0.05) frequency of MN was observed among farmers as compared to control based on gender and smoking status. As for BMI classification, a significant difference (*p* < 0.05) in the frequency of MN was only seen among normal and pre-obese of inter group comparison. A significant increase in MN frequencies (*p* < 0.05) was also detected for different duration of employment among farmers as compared to control. No significant difference was seen in the frequency of BNu for inter group comparison.

The correlation between MN and BNu frequencies with socio-demographic factors (age, years of employment, BMI) and duration of exposure to pesticides and fertilizers were studied. As shown in Table 7, a significant (*p* < 0.05) and positive correlation between the duration of fertilizers exposure and frequencies of BNu (*r* = 0.37, *P* = 0.02) and MN were (*r* = 0.42, *P* = 0.001) observed. However, no other correlation of various confounding factors on MN and BNu were observed (Table 7). The frequencies of BNu and MN show a weak positive correlation with the duration of pesticides exposure and the correlation was not significant (*p* > 0.05).

**Table 7:** Correlation between the frequencies of MN and BNu with the exposure to pesticides and fertilizers

	Frequency of MN	Frequency of BNu
	(*n* = 39)	(*n* = 39)
Duration of pesticides exposure (hours)	0.15	0.28
	(*p* = 0.37)	(*p* = 0.16)
Duration of fertilizers exposure (hours)	0.42	0.37
	(*p* = 0.001)*	(*p* = 0.02)*

*p < 0.05

## Discussion

Farmers who are exposed to pesticides are at greater risk for cytogenetic damage. To date, a number of studies have been carried out on the health status of pesticide-exposed populations from different countries to elucidate the risk associated with pesticide-induced cytogenetic damage [7–10]. However, no definitive conclusions could yet be established on the association of these factors. Up to our knowledge, there have been no reports in Malaysia concerning the issue, suggesting the need for such studies to be conducted. In the present study, a preliminary investigation on cytogenetic damage among pesticides and fertilizers-exposed farmers from two rural areas of North East Malaysia, namely Bachok and Pasir Puteh was carried out. Cytogenetic damage was determined by the frequency of micronucleus (MN) and binucleus (BN) of isolated buccal epithelial cells. In order to identify the confounding factors that may contribute to cytogenetic damage, information on demographics, pesticide exposure and PPE application were also gathered.

The results obtained in this study showed a significant increase of MN frequencies in farmers when compared to controls. The results obtained for MN is in agreement with previous studies which observed higher frequency of MN among pesticide-exposed farmers than the non-exposed group [31–33]. Although some studies reported no significant increase in the MN frequencies in pesticide-exposed farmers [25, 30, 34], the presence of MN as indicators for cytogenetic damage has been acknowledged as a useful biomonitoring tool in populations occupationally exposed to pesticides [35].

In contrast to MN results, BNu frequencies revealed no significant difference between the two studied groups. The result could be influenced by the presence of uncontrolled exogenous factors such as environmental influence that contributed in the evaluation of cytogenetic damages [36]. Moreover, a lack of comparable data in the literature concerning BNu frequencies as compared to MN indicated the unresolved role of BNu as a marker for nuclear abnormalities associated with pesticide toxicity.

Pesticides exposure has been linked to a number of human health effects [37, 38]. Thus, an attempt was made to identify the possible chronic diseases and symptoms that are commonly associated with pesticide exposure among farmers. The number of farmers with reported hypertension, asthma, gastritis, insomnia, runny nose and coughing were higher as compared to the control group. Moreover, more than 50 % of farmers claimed frequent experience of eye irritation, blurred vision and numbness, suggesting a possible association to pesticide exposure. However, due to lack of medical history and nutritional status data, direct association of the manifested symptoms with pesticide exposure could not be established. Lack of PPE application during pesticide handling however can be one of the contributing factors for the reduced quality of health among the farmers.

The use of appropriate PPE should give a significant preventive impact in pesticide exposure among farmers. Significant increase in cytogenetic damage among farmers with no or little protective clothing during pesticide usage has been reported [39–41]. The usage of PPE, as observe in this study can be regarded as unsatisfactory. Of the 7 PPE assessed, only 4 of the PPE (safety helmets, long sleeved shirt, long pants and nose mask) were used by the majority of the farmers. Furthermore, the use of eye goggles, gloves and boots were considered low with a significant number of farmers who claimed that they never used these PPE throughout their farming activities which put them at greater risk of percutaneous exposure to pesticide. In a study conducted by Lander et al. [32], remarkable cytogenetic effects were noted in workers who did not use gloves, indicating the importance of PPE usage. Although the humid and hot climatic conditions in Malaysia could be the factor that limits the usage of full protective clothing [42, 43], continued effort to increase awareness among farmers on potential pesticide hazards and the importance of PPE usage can be useful to minimize pesticide related hazards at the workplace.

The presence of nuclear anomalies can be influenced by various confounding factors which include the exogenous (alcohol, diet, smoking and pesticide application) and endogenous (age and gender) factors [44–47]. Some studies have reported a positive age effect on MN frequencies among workers [33, 48]. However, in this study, no significant differences were seen on the frequencies of MN and BNu for both farmers and controls, which is in agreement to previously reported studies [34, 49].

Apart from age, the frequency of MN is also influenced by gender. Higher MN frequency in women than in men was commonly observed in populations without exposure to any physical or chemical agents, with X chromosome micronucleation being shown to contribute to the increase of MN frequency in women [44]. In this study however, no significant differences for the frequencies of MN and BNu between male and female subjects were observed, though increased MN frequency in female farmers were observed and is in accordance with a previous report [49].

Level of education may affect the level of knowledge and awareness on the safety of pesticide handling [50]. A previous study reported that the level of education with at least high school education is required for farmers to show a positive perception on the usefulness of PPE application [51]. In a study conducted among Brazilian farmers, the inability to understand the information displayed on products’ labels led to increased exposure to pesticides [52]. A survey conducted on 61 randomly selected fruit-growers in a specific area of Turkey [53] revealed that pesticide practices were influenced by a number of characteristics, with those who consider pesticides as harmful consisted of farmers who are younger, better educated and have less experience in fruit-growing. Our findings indicated that almost half of the farmers did not have formal education beyond the secondary education level. Therefore, levels of education may affect the perception they may have towards the harmfulness of the pesticides.

Tobacco smoke is a known genotoxicant and evidences on its ability to induce DNA damage have been comprehensively studied [45]. However, in this study the influence of smoking on cytogenetic damage showed no significant correlation as observed in the frequencies of MN and BNu between smokers and non- smokers within the pesticide exposed group. Similarly, the effect of smoking was not found in MN and BNu frequencies within the control group. The lack of association observed between the frequencies of studied nuclear anomalies and tobacco smoke exposure has been reported previously [30, 54]. The effect of tobacco smoke exposure on genotoxicity is influenced by type of cigarettes and frequencies of smoking [54] both of which were not evaluated in this study. Thus, future studies focusing on gathering comprehensive information on smoking habits among farmers can provide more definitive correlation and conclusive evidence.

Duration of farming experience provides information on the degree of exposure to pesticide among farmers [4]. In a review by Bolognesi [14], occupational exposure to pesticide enhances genotoxic damage in a dose-dependent manner. In this study, higher frequencies of MN were observed among farmers with farming experience of 41–50 years as compared to those with shorter durations of farming experience. However, the observed difference was not statistically significant. It appears that duration of farming activities demonstrated no significant influence on the frequency of MN and BNu among farmers, which is in agreement with other reported studies [55, 56]. To date, studies on the association between pesticide exposure and frequency of MN and BNu are inconclusive. Eastmond [57] have indicated that the persistence of chromosomal damage is short-lived for acute exposure while Scarpato et al. [58] noted that damage may be reduced during low exposure periods for seasonal workers. However, increased chromosomal damage associated with duration of agricultural employment was reported by a number of studies [17, 58, 59]. The conflicting results from studies on pesticide-related cytogenetic damage were influenced by many factors such as the working environment, the studied populations, the type of exposure to pesticides such as seasonal application of pesticides, types of pesticide mixtures and the use of PPE [19, 60, 61]. Moreover, Bull et al. [4] has stated in a review that chromosomal damage itself is regarded as transient and sampling time play an important role on determining the accuracy of the data. Sampling should be done within 2 days following acute or cessation of chronic exposure to overcome false negative results. However, we have not considered this possibility which could contribute to our non-significant finding.

Assessments of correlation between MN and BNu frequencies with duration of pesticides and fertilizers exposures (hours) were also determined. The length of pesticide exposure had a positive correlation with BNu and MN frequencies, although the association was not statistically significant. However, there is a significant and positive correlation between the duration of fertilizers exposure and frequencies of BNu and MN, suggesting a potential workplace hazard faced by the farmers. However, considering that this is a cross sectional study which has its limitation, no cause and effect could be established from the findings. Thus, the direct effects of pesticide and fertilizer exposures on the induction of nuclear abnormalities among exposed farmers could not be concluded at this stage of studies and warrant further investigation.

## Conclusion

From our results, the pesticides and fertilizers usage in farming can contribute to the induction of cytogenetic damage. The role of age, sex, smoking and years of agriculture activities in MN and BNu frequencies were not evidenced and the result could be affected by small sample size. Thus, future studies would likely require assessment of much larger sample sizes to better characterize the effect of confounding factors in cytogenetic damage. It was notable that female farmers can be considered to be at higher risk for cytogenetic damage related to pesticide exposure as compared to male farmers. Increased exposure to fertilizers and pesticides and inappropriate use of PPE can be associated with increased cytogenetic damage. This finding can serve as a foundation for future biomonitoring studies in Malaysia.
